# TB therapeutic drug monitoring – analysis of opportunities in Romania and Ukraine

**DOI:** 10.5588/ijtld.22.0667

**Published:** 2023-11-01

**Authors:** I. Margineanu, T. Butnaru, F. Gafar, D. Baiceanu, R. Dragomir, I. Semianiv, F. Mihaltan, I. Munteanu, B. Mahler, L. Todoriko, S. Margineanu, O. Akkerman, Y. Stienstra, J-W. C. Alffenaar

**Affiliations:** 1 Department of Clinical Pharmacy and Pharmacology, University Medical Centrum Groningen, University of Groningen, Groningen, The Netherlands; 2 Prof Dr Marius Nasta Institute for Pneumology, Bucharest, Romania; 3 Unit of Pharmacotherapy, Epidemiology and Economics, Groningen Research Institute of Pharmacy, University of Groningen, Groningen, the Netherlands; 4 Chernivtsi TB Expertise Centre, Bukovinian State Medical University, Chernivtsi, Ukraine; 5 Department of Computer and Data Sciences, Ion Ionescu de la Brad Iasi University of Life Sciences, Iasi, Romania; 6 Department of Pulmonary Diseases and Tuberculosis, University Medical Center Groningen, University of Groningen, Groningen; 7 Tuberculosis Center Beatrixoord, University Medical Center Groningen, University of Groningen, Haren; 8 Department of Internal Medicine/Infectious Diseases, University Medical Center Groningen, University of Groningen, Groningen, The Netherlands; 9 Department of Clinical Sciences, Liverpool School of Tropical Medicine, Liverpool, UK; 10 Faculty of Medicine and Health, School of Pharmacy, and; 11 Sydney Institute for Infectious Diseases, The University of Sydney, Sydney, NSW; 12 Westmead Hospital, Sydney, NSW, Australia

**Keywords:** personalised, patient-centred, hospital, diagnosis, treatment

## Abstract

**INTRODUCTION:**

Therapeutic drug monitoring (TDM) could improve TB treatment outcomes by avoiding drug toxicity or underdosing. In this study, we describe the patient burden in three TB centres in Romania and Ukraine with a TDM indication, as per the current guidelines, in order to estimate the feasibility of implementing TDM.

**METHODS:**

A retrospective multi-centre study was conducted at the Iasi Lung Hospital (Iasi, Romania), Bucharest Marius Nasta Institute (Bucharest, Romania) and Chernivtsi TB Centre (Chernivtsi, Ukraine) in adult hospitalised TB patients.

**RESULTS:**

A total of 927 participants were admitted, of whom 37.8% had at least one indication for TDM, the most frequent being slow response to TB treatment (202/345, 58.6%); 55.5% had at least one cavity present on chest X-ray. Patients with a TDM indication stayed in the hospital for a median of 67 days and took on average 2 months more to reach a successful TB outcome.

**CONCLUSION:**

TDM could be a valuable tool to improve management of selected TB patients. The decision on whether to perform TDM is often delayed by 2 months due to waiting for culture results after treatment initiation. A randomised control trial should be performed in order to define TDM’s precise role in TB therapy.

## Abstract (fr)

**CADRE:**

Le suivi thérapeutique des médicaments (TDM) pourrait améliorer les résultats du traitement de la TB en évitant la toxicité des médicaments ou le sous-dosage. Dans cette étude, nous décrivons la charge de travail des patients dans trois centres antituberculeux de Roumanie et d’Ukraine avec une indication de TDM, conformément aux directives actuelles, afin d’estimer la faisabilité de la mise en œuvre du TDM.

**MÉTHODES:**

Ceci est une étude rétrospective multicentrique a été menée à l’hôpital pulmonaire de Iasi (Iasi, Roumanie), à l’Institut Marius Nasta de Bucarest (Bucarest, Roumanie) et au centre antituberculeux de Chernivtsi (Chernivtsi, Ukraine) auprès de patients adultes hospitalisés pour une TB.

**RÉSULTATS:**

Au total, 927 participants ont été admis, dont 37,8% avaient au moins une indication de TDM, la plus fréquente étant une réponse lente au traitement de la TB (202/345; 58,6%); 55,5% avaient au moins une cavité présente à la radiographie thoracique. Les patients pour lesquels le TDM était indiqué sont restés à l’hôpital pendant une durée médiane de 67 jours et ont mis en moyenne 2 mois de plus pour obtenir une issue favorable à la TB.

**CONCLUSION:**

Le TDM pourrait être un outil précieux pour améliorer la prise en charge de certains patients tuberculeux. La décision d’effectuer ou non un TDM est souvent retardée de deux mois en raison de l’attente des résultats de la culture après le début du traitement. Un essai contrôlé randomisé devrait être réalisé afin de définir le rôle précis du TDM dans le traitement de la TB.

TB remains one of the most globally impactful infectious diseases, with a mortality of 1.3 million in 2020. Global average treatment success rates are 86% for drug-susceptible TB (DS-TB) and 59% for multidrug-resistant TB (MDR-TB).^1^ Suboptimal treatment success rates have been linked to several factors, either those related to case severity or to the treatment itself. In the former category, TB management can be complicated by various factors, such as comorbidities (e.g., HIV or diabetes mellitus [DM]), differences in lifestyle (e.g., alcohol consumption, smoking) and TB severity (e.g., the presence of cavities). In the latter, the already complex and lengthy treatment can be further impaired by low or high drug exposures.^2^ In this context, therapeutic drug monitoring (TDM) is a promising tool, as it can help with clinical decision-making by offering accurate measurements of drug concentrations allowing clinicians to make informed decisions on drug dosing.^3^ Several global guidelines, including the WHO guideline for DR-TB, the American Thoracic Society Guidelines for TB and The Union’s Clinical Standards recommend TDM for patients at risk of/or experiencing either drug toxicity, slow response to treatment, treatment failure or acquired resistance.^4^–^7^ Those living with HIV, those diagnosed with advanced kidney disease, DM and patients with DR-TB are considered at-risk patients.

Romania has the highest TB burden within the European Union (EU), accounting for a fourth of all TB cases in the EU,^8^ while Ukraine, bordering Romania to the north, is one of the top 30 countries with the highest TB burden, especially for MDR-TB.^9^ Guidelines in both countries recommend admitting patients with TB disease at least for treatment initiation, ideally until culture negativity.^10^ Due to its high implementation costs and the lack of familiarity among clinicians and national stakeholders with this technique, TDM is currently not part of routine care in these settings. Furthermore, despite a large body of knowledge on the optimum plasma concentrations of TB drugs,^11^ there are gaps in the information about implementing TDM in resource-scarce settings at the community level.^3^ To facilitate the future implementation of TDM strategies, it is crucial to obtain estimates of patients with a TDM indication. These estimates will serve as a fundamental basis for conducting comprehensive cost-effectiveness studies.

In this study, we describe the patient burden in three TB centres in Romania and Ukraine with a TDM indication as per the current guidelines.

## METHODS

### Design and setting

A retrospective multi-centre study was conducted in the Iasi Lung Hospital (Iasi, Romania), Bucharest Marius Nasta Institute (Bucharest, Romania) and Chernivtsi TB Centre (Chernivtsi, Ukraine).

The Iasi Lung Hospital is the regional TB centre for the north-eastern region of Romania. The Marius Nasta Institute is the national Romanian TB expertise centre. Chernivtsi TB Centre, a specialist TB centre, is located in the Chernivetska Oblast, in south-western Ukraine. Inclusion criteria were hospitalised adult patients (aged >18 years) diagnosed with TB of any form, who initiated treatment between 1 January 2019 and 31 December 2020, and had both renal function and liver enzymes measured at least once during or at start of TB treatment. Hospitalised TB patients in all centres have documented diagnosis data, known HIV status and chronicled hospitalisation data. Other comorbidities were either documented (e.g., glycated haemoglobin levels for DM) or derived from medical history.

The study was approved by the ethics committees of Iasi Lung Hospital (Iasi, Romania), Bucharest Marius Nasta Institute (Bucharest, Romania) and Chernivtsi TB Centre (Chernivtsi, Ukraine) (Iasi: #5483/2021; Bucharest: #10592/2019,; Chernivtsi: #234/2021).

### Data collection and storage

Patient- and disease-related data were extracted from medical records and captured in Research Electronic Data Capture (REDCap) v11.0.3 (Vanderbilt University, Nashville, TN, USA), a secure web-based platform. Data extraction was verified by another investigator. Data collection mistakes were corrected and again verified by another investigator. After completion of data collection, IM performed a final random check of all collected data. Data collection included general demographics, smoking and alcohol consumption, and known comorbidities at the time of TB diagnosis. Diagnosis data included symptoms, chest X-ray interpretation and results from direct microscopy of sputum and culture of sputum or other biological material. Baseline admission data included treatment programme and hepatic and renal laboratory tests at baseline. Hospitalisation data included laboratory parameters, adverse events (AEs) and management of hepatic or renal toxicity. TB treatment outcome was collected using WHO-defined TB outcomes (time to culture conversion in months, total duration of treatment, treatment success, loss to follow-up, death).^1^

For each patient, indications for TDM were collected. TDM indications as listed in the guidelines were as follows:^12^,^13^ slow response to TB treatment (culture conversion beyond 2 months for DS-TB and 4 months for MDR-TB), severe gastro-intestinal abnormalities (severe gastroparesis, short bowel syndrome, chronic diarrhoea with malabsorption), impaired renal clearance (defined as glomerular filtration rate <60 ml/min/1.73 m^2^, CKD-EPI (Chronic Kidney Disease Epidemiology Collaboration) or acute kidney failure (drop in estimated glomerular filtration rate [eGFR] of at least 50%), comorbidities (HIV infection, DM type 2) and use of second-line TB medication. Descriptive statistics were used to characterise the data set and the programme used was IBM SPSS v.27 (IBM, Armonk, NY, USA). All definitions of TB cases and outcomes follow the WHO framework.

## RESULTS

### General characteristics

Between 1 January 2019 and 31 December 2020, there were 927 TB patients admitted in all three centres. After excluding 19 patients due to the unavailability of documented follow-up data, 912 patients were included in the analysis: 378 in Iasi, 507 in Bucharest and 154 in Chernivtsi. The median age was 47 years (interquartile range [IQR] 35–61); 67.1% were male, 58.1% lived in rural areas, 48.1% were current smokers and 60.8% were current alcohol consumers.

The most frequent comorbidities were cardiovascular (e.g., hypertension, heart failure), in 28.3% of patients, followed by chronic obstructive pulmonary disease (COPD; 14.7%) and DM (6.4%). HIV was present in 2.3% of patients. The majority (838/912, 91.9%) had DS-TB, 20/912 (2.2%) had mono-resistant TB, 18/912 (2%) had MDR-TB and 36/912 (3.9%) had pre-extensively drug-resistant (XDR-TB). The majority of patients with rifampicin-resistant TB (RR-TB) were from Ukraine (54 RR-TB in Ukraine vs. 20 in Romania).

More than a third of the patients (345/912, 37.8%) had at least one indication for TDM, 244/345 (70.7%) of whom had only one indication for TDM, 65 (18.8%) had two and 36 had three or more (10.4%). The most frequently observed indication for TDM was slow response to TB treatment (202/345, 58.6%), followed by administration of second-line medication, including patients with DS-TB who were intolerant to one of the first-line drugs (92/345, 26.1%). Of the 92 participants, 72 (97.3%) had DR-TB (Figure).

**Figure:**
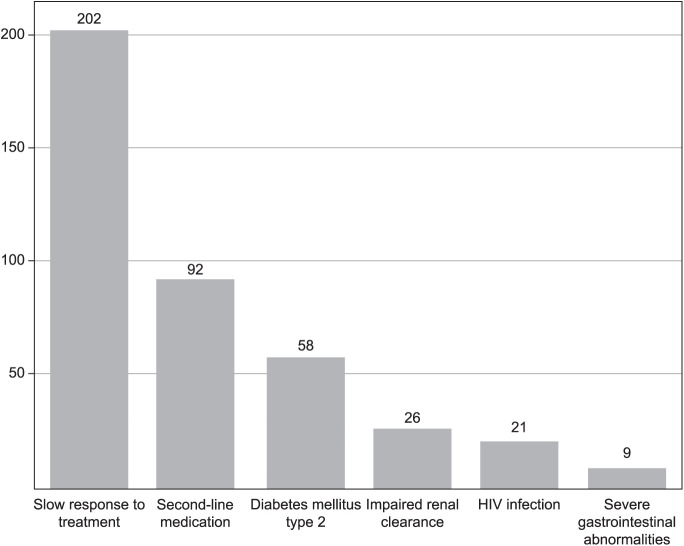
Number of patients who had guideline indications for TDM. Slow response to tuberculosis treatment - culture conversion beyond month 2 for drug-susceptible TB and 4 months for drug-resistant TB despite adherence; severe gastro-intestinal abnormalities - severe gastroparesis, short bowel syndrome, chronic diarrhoea with malabsorption; Impaired renal clearance – CKD at least Stage 4 (eGFR < 30 ml/min/1.73 m^2^, CKD Epidemiology Collaboration) or acute kidney failure (drop in eGFR of at least 50%). TDM = therapeutic drug monitoring; CKD = chronic kidney disease; eGFR = estimated glomerular filtration rate.

### Description of the population with TDM indication

The three clinical sites were different in their proportion of patients with TDM indication, with Chernivtsi, Ukraine, having 125 patients with TDM indication out of 154 (81%), and sites in Romania having respectively 29.4% and 28.7% for Iasi and Bucharest. Sex, age or living situation (rural, urban, homeless) between the groups were comparable. Most (82%) were new TB patients, with the remainder being relapse cases. The majority had pulmonary TB (79.4%), with 9% presenting with a combination of lung and pleural TB. Regarding pulmonary TB diagnosis, 59.1% had bilateral involvement of the lungs, and 55.5% had at least one cavity present on pulmonary chest X-ray; 26.4% had sputum with >9 bacilli/field (+++) and 35.4% had culture result of >100 colonies (statistically significantly more than the group without TDM indication).

Patients with TDM indication stayed in the hospital for a median of 67 days (IQR 33.5–98), in contrast with patients without a TDM indication who stayed a median of 25 days (IQR 23–64). Increased liver enzymes were observed in 31.9% of the patients with TDM indication, with 7.3% having a >5 times elevation in aspartate transaminase levels. Overall, 19.4% had abnormal eGFR during hospitalisation, with 2.3% having acute kidney injury (defined by a decrease in eGFR of >50%). The time recorded for abnormal values – both hepatic and renal – to improve and/or normalise was a median of 33 days (IQR 19–60), and 17.6% of patients with abnormal values did not experience a significant improvement, despite clinical measures taken, including supportive measures and changes in medication.

Side effects were reported in 24.6% of patients with TDM indication, the most frequent being gastro-intestinal (e.g., nausea) in 7.8% of cases, while 2% presented with a severe allergic reaction.

Clinical measures taken to improve laboratory parameters or mitigate side effects included supportive treatment (3.5%), lowered dosages of TB drugs (4.4%), pausing TB medication for more than 7 days (4.6%). The clinical decision to stop and/or switch TB medication was made for 15.4% of patients with TDM indication (vs. for 0.7% in patients without TDM indication). The most frequently involved drugs were pyrazinamide, which was replaced by a fluoroquinolone (FQ), either levofloxacin or ofloxacin (8/25); the remainder of the cases on other first-line drugs were switched to an FQ, and in five cases to a combination drug, including an injectable drug (amikacin or kanamycin).

In terms of WHO TB outcomes, 86.6% of patients with TDM indication achieved treatment success; in patients without TDM indication, this was 88%. Death was recorded in 5.7% of patients with TDM indication and 6.7% of patients without TDM indication. Treatment duration was a median of 2 months longer for patients with TDM indication (8 months, interquartile range [IQR] 6–10 vs. 6 months, IQR 6–8) (Table).

**Table:** Characteristics of patients with TDM indication

Characteristics	Patients with TDM indication (*n* = 345) *n* (%)
Study site	
Iasi	111 (29.4)
Bucharest	109 (28.7)
Chernivtsi	125 (81.2)
Demographics	
Age, years, median [IQR]	48 [37–60]
Male sex	232 (67.2)
BMI, kg/m^2^, median [IQR]	20.8 [18.8–22.9]
Living conditions	
Urban	130 (37.7)
Rural	208 (59.1)
Homeless	9 (2.6)
Lifestyle	
Current smoker	175 (51)
Former smoker	39 (11.3)
Currently consuming alcohol	187 (65.8)
Comorbidities	
Asthma	0 (0)
COPD	70 (20.3)
Cancer	7 (2)
Cardio-vascular	136 (39.4)
Gastro-intestinal (including chronic gastritis, ulcer)	72 (20.9)
Cirrhosis of the liver	8 (2.3)
Chronic kidney disease	12 (3.5)
Number of comorbidities, mean ± SD	1.16 ± 0.99
TB characteristics	
New case	283 (82)
Rapid relapse (previous TB under 2 years ago)	16 (4.6)
Number of symptoms at initial presentation, median [IQR]	4 [2–5.5]
Anatomical site	
Lung	274 (79.4)
Pleural TB without lung involvement	14 (4.1)
Lung and extrapulmonary	31 (9)
Extrapulmonary other than pleura	26 (7.5)
Chest X-ray	
Pulmonary TB with both lungs involved	246 (71.3)
At least one cavity present	191 (55.5)
Fibrosis present	38 (11)
Sputum	
Negative	55 (15.9)
Paucibacillar	35 (10.1)
+ (10–99 bacilli/100 fields)	46 (13.3)
++(1–9 bacilli/field)	89 (25.8)
+++(>9 bacilli/field)	91 (26.4)
Culture	
Negative	12 (3.5)
1–9 colonies	57 (16.5)
10–100 colonies	126 (36.5)
>100 colonies	122 (35.4)

TDM = therapeutic drug monitoring; IQR = interquartile range; BMI = body mass index; COPD = chronic obstructive pulmonary disease; SD = standard deviation.

## DISCUSSION

In this retrospective study conducted at three centres in Romania and Ukraine, where TDM is not currently part of routine practice, it was observed that over a third of the patients with TB disease would have a clear indication for TDM if this strategy were to be implemented. The most common criterion for TDM indication was a slow response to treatment. There were differences in the percentage of patients with TDM indication per centre: in Ukraine, 81.2% of hospitalised patients in 2019 had a TDM indication, whereas in Romania, this was 29.4% and 28.7%. This reflects, for example, the differences in the admitted patient populations, with Ukraine having the majority of the RR-TB cases. Furthermore, this could be attributed to differences in the base population of admission, where Ukraine has a lower income level than Romania, leading to a higher prevalence of RR-TB cases. In a resource-constrained setting, the decision to treat patients on an ambulatory basis is possibly more common, although this may deviate from national guideline recommendations. To note, the proportion of patients who achieved treatment success was comparable to patients without TDM indications, but the treatment duration was longer, likely due to guidelines recommending prolonged treatment based on delayed culture conversion.

Our study findings indicate that more than a third of patients (37.8%) were eligible for TDM according to the guidelines. Although the study sites were located in middle-income countries, another study with a similar methodology performed in Australia, showed that a similar proportion of patients (35%) were eligible for TDM.^14^ We speculate that this indicates that a significant proportion of patients would be eligible for TDM, irrespective of the setting. However, this observation also presents potential challenges when considering the feasibility of implementing TDM.

In our study, guideline TDM indicators may have led to the selection of clinically worse patients. This is congruent with current knowledge that patients with comorbidities and/or more TB symptoms are at higher risk of suboptimal treatment outcome or of developing adverse drug-related events. More than half (58.6%) of patients in our study were included due to the slow response to treatment, compared to only 9% in the previously mentioned Australian study, in which patients were more often selected based on comorbidities (e.g., DM). Factors influencing time to culture conversion in HIV-negative DS-TB patients include the presence of DM and extensive disease, smoking and alcohol consumption.^15^ In case of MDR-TB patients, one study concluded that the main risk factors for delayed culture conversion are high smear grade, smoking, alcohol consumption and ofloxacin resistance.^16^ In our study, patients with TDM indication consumed alcohol more frequently, had more comorbidities and tended to have more severe disease, with higher smear grade and worse culture results, bilateral lung involvement; also, a greater number had cavitary disease. However, as culture conversion depends on various clinical parameters, it should not be used alone to predict TB treatment effectiveness.^17^ Furthermore, in an international survey TB experts said that only 7% of those who perform TDM use slow response to treatment as a criterion for initiating TDM.^18^

Guidelines recommend TDM if second-line drugs are used in the treatment programme, regardless of resistance status. Indeed, out of 92 patients who received second-line drugs, 20 had DS-TB and were treated with either an FQ and/or an injectable drug. The evidence seems to indicate that FQs might be underdosed in patients with DR-TB;^2^ furthermore, rifampicin could affect moxifloxacin drug concentrations in DS-TB patients.^19^–^21^ TDM could help clinicians in both these cases. More robust studies are needed to clarify the benefits of TDM for FQ use in DS-TB.^22^

Patients with TDM indication were hospitalised for a median duration of more than 2 months, while patients without TDM indications were discharged after 35 days. The national TB guidelines of Romania and Ukraine recommend hospitalisation until at least the patient is sputum-negative on microscopy, preferably until culture negativity.^23^ This indicates that patients with more severe disease, comorbidities and side effects tend to have prolonged hospital stays, primarily influenced by their specific medical condition. As previously mentioned, these findings strongly align with the TDM indications. Nevertheless, despite WHO TB outcomes not being statistically significantly different between the groups, patients with TDM indication stayed on treatment for a median of 2 months longer than patients without TDM indication, indicating that to achieve the same outcomes, more effort and resources had to be provided. Based on the higher additional costs per patient for extended hospitalisation in Romania (€4,363 euros) and Ukraine (€1,109 euros),^24^,^25^ there is a potential economic benefit in addition to clinical benefit associated with treatment optimisation of these hospitalised patients. The implementation of TDM may be associated with higher costs, both in terms of the dedicated equipment required (e.g., high-performance liquid chromatography [HPLC]) and related consumables (assays) and the need for trained personnel. The willingness to implement TDM would be facilitated by additional evidence in favour of cost-effectiveness. The evaluation of the impact of TDM should include strategies for identifying patient categories, facilitating data collection and analysis, e.g., from dried blood spots and saliva samples, and optimising the use of HPLC tools. By employing various TDM methods, such as dried blood spot and saliva samples, and strategically prioritising specific patients, TDM emerges as a potentially cost-effective intervention, especially in TB hospitals attached to university centres, which would have access to a HPLC machine from other departments. This would help to overcome one of the most important barriers to TDM implementation, which is the perceived large costs associated with this technique.^18^ Robust economic studies based on randomised clinical trials evaluating TDM are needed to clarify the cost-effectiveness of implementing TDM, especially in lower-income countries with high TB burdens.

### Strengths and limitations

This large-scale retrospective study was conducted in three centres located within the European region, with a particular focus on Romania, which accounts for approximately one fourth of all TB cases reported in the EU/EEA (European Economic Area), and Ukraine, which has the fifth highest number of confirmed cases of XDR-TB in the world. The inclusion of Romania and Ukraine therefore adds significant relevance, given their substantial contribution to the TB burden in the European setting. Despite the retrospective nature of this study performed where TDM is not yet routinely implemented, data indicate potential benefits in implementing TDM in these settings, as evidenced by the number of patients with a TDM indication. Our findings suggest that the decision to implement TDM should be considered on a centre-by-centre basis.

## CONCLUSION

This study, conducted in a high TB burden setting, shows that more than a third of TB patients are eligible for TDM according to current guidelines, most of whom are included because of slow response to treatment. In this context, the decision to perform TDM (Therapeutic Drug Monitoring) is often delayed due to the necessity of waiting for culture results, which are typically collected two months after the initiation of treatment. An alternative approach would be to start TDM after 1 or 2 weeks of treatment in those with risk factors for sub-optimal drug exposure,^5^ e.g., patients with cavities, which would impair drug effectiveness due to the drug’s poorer penetration in the cavities; however, this decision should be mindful of the resources available.^19^,^20^ The decision to implement TDM in a particular setting should take into account patient profiles, cost-effectiveness and potential benefits; a randomised controlled trial to further investigate the potential TDM opportunities in low-resource settings is thus warranted.
